# Lingual Thyroid: Diagnostic and Therapeutic Insights. A Case Report

**DOI:** 10.2174/0118715303470877260513192103

**Published:** 2026-06-23

**Authors:** Lingling Song, Jing Yang, Huangruici Zhang, Lin Luo, Yu Zhang, Fengfeng Jia, Min Guo

**Affiliations:** 1 Department of Otorhinolaryngology, The First Affiliated Hospital of Kunming Medical University, Kunming, China

**Keywords:** Lingual ectopic thyroid, hypothyroidism, thyroid scintigraphy, thyroid hormone replacement therapy, differential diagnosis, multimodal examinations

## Abstract

**Introduction:**

Ectopic Thyroid (ET) predominantly locates at the tongue base (~90%) but is easily misdiagnosed due to non-specific symptoms. Most cases are complicated with hypothyroidism, and blind surgery may lead to permanent hypothyroidism. This case report is a typical yet easily missed presentation, providing practical diagnostic and therapeutic references for clinicians facing similar unexplained pharyngeal symptoms.

**Case presentation:**

A 44-year-old female presented with unexplained pharyngeal foreign body sensation and was initially suspected of a tongue base mass. Multimodal examinations (fibrolaryngoscopy, neck CT, thyroid ultrasound, scintigraphy, and thyroid function tests) revealed a semicircular tongue base bulge, absent normal thyroid tissue in the anatomical site, and decreased thyroxine (T4), confirming lingual ET. She received thyroid hormone replacement therapy and was followed up regularly.

**Conclusion:**

Clinicians should consider lingual ET in patients with unexplained pharyngeal symptoms. Multimodal examinations are essential for accurate diagnosis, and individualized treatment (prioritizing hormone replacement over blind surgery) is critical to avoid irreversible hypothyroidism. Standardized management ensures a favorable prognosis, highlighting the value of differential diagnosis for atypical head and neck symptoms.

## 1 INTRODUCTION

Ectopic Thyroid (ET) is a clinical disorder characterized by abnormal thyroid gland development. The first reported case involved a neonate with ET, who died of severe dyspnea and asphyxia 16 hours after birth; eventually, the cause of death was confirmed to be lingual thyroid [1]. ET is relatively rare in clinical practice. Due to the lack of specificity in its location and clinical manifestations, it is prone to misdiagnosis. Clinicians should fully integrate medical history and auxiliary examinations to make a clear diagnosis and avoid misdiagnosis and mistreatment. This article reports a case of lingual thyroid and collects and summarizes the data of ET cases reported in the literature, aiming to provide a reference for the rational diagnosis and treatment of lingual thyroid.

### 1.1 Case Report

A 44-year-old female farmer presented with a self-reported foreign body sensation in the pharynx without obvious inducement in April 2025, and she initially sought medical attention at a local hospital. During the disease course, she had no accompanying symptoms such as sore throat, dry throat, itchy throat, fever, cough, dyspnea, or dysphagia. Desiring surgical treatment, she then visited the Department of Otorhinolaryngology at the First Affiliated Hospital of Kunming Medical University. The outpatient fibrolaryngoscopy revealed a semicircular bulge at the base of the tongue, and she was admitted to the hospital with a diagnosis of “tongue base mass” for planned surgery. After admission, she completed routine preoperative examinations, Computed Tomography (CT), ultrasonography (B-ultrasound), thyroid function tests, and thyroid scintigraphy (SPECT-CT). During the disease course, the patient had a good mental state and appetite, normal defecation and urination, and no significant weight loss. One year prior to this presentation, she had undergone hernia surgery with adequate postoperative recovery and no complications. Her past medical history was otherwise unremarkable. She denied any family history of genetic disorders, as well as any personal history of smoking or alcohol use.

Physical Examination: The pharynx was free of congestion, and bilateral tonsils were not enlarged. There was no deformity of the bilateral auricles; the bilateral external auditory canals were unobstructed, and the bilateral tympanic membranes were intact with clear landmarks. The external nose had no deformity, the nasal septum was not deviated, and no obvious abnormal secretions were found in the bilateral nasal cavities. The patient was in stable general condition with stable vital signs.

Laboratory Examinations: Routine blood tests, coagulation function, hepatic and renal function, fasting blood glucose, blood lipids, serum potassium/sodium/chloride, as well as tests for hepatitis B, hepatitis C, syphilis, and Human Immunodeficiency Virus (HIV), all showed no abnormalities. Thyroid function test results indicated hypothyroidism: thyroxine (T4) was 63.60 nmol/L (normal range: 66-181.0 nmol/L), free thyroxine (FT4) was 10.53 pmol/L (normal range: 12.0-22.0 pmol/L), and reverse triiodothyronine (rT3) was 0.37 nmol/L (normal range: 0.54-1.46 nmol/L).

Imaging Examinations: For fibrolaryngoscopy (as shown in Fig. **1A**), a semicircular bulge with a relatively smooth surface was observed at the base of the tongue. Lymphoid follicular hyperplasia was present in the posterior pharyngeal wall and posterior tongue base. The epiglottis was smooth, the bilateral pyriform sinuses were symmetrical; the bilateral vocal cords were smooth with normal mobility, and glottic closure was adequate. For neck CT (as shown in Fig. **1B**), an isodense mass shadow was seen in the midline of the tongue base, with relatively clear boundaries and a size of approximately 17×14×18 mm (anteroposterior × left-right × superoinferior); it showed obvious enhancement on contrast-enhanced scan, accompanied by multiple tortuous small strip-like vascular shadows around it, while the oropharyngeal cavity at the level of the lesion was narrowed, the epiglottis was not clearly visualized, the epiglottic vallecula disappeared, and no definite thyroid gland was shown. For thyroid and cervical lymph node ultrasound (as shown in Fig. **2A**), no definite thyroid structure was detected in the original thyroid area, but a slightly hypoechoic nodule was found in the anatomical region of the left thyroid lobe, with a size of approximately 0.30×0.26 cm, regular shape, and clear boundaries. For the SPECT-CT image (as shown in Fig. **2B**), no thyroid structure was displayed in the thyroid bed area, while a patchy, slightly hyperdense shadow was observed in the midline of the tongue base, with a size of approximately 2.3 cm×1.9 cm×2.8 cm and increased iodine uptake function, suggesting the possibility of ET.

Diagnosis and Treatment Outcomes: Based on the patient's medical history and ancillary investigations, a diagnosis of lingual ectopic thyroid with associated hypothyroidism was established. Following multidisciplinary consultation with the Departments of Endocrinology and Thyroid Surgery, surgical intervention was deferred in favor of initiating Thyroid Hormone Replacement Therapy (THRT) alongside a plan for regular follow-up. The patient was started on oral levothyroxine sodium at a dose of 25 µg once daily. She demonstrated good treatment adherence, confirmed through regular follow-up visits and medication records. No adverse effects such as palpitations, insomnia, or significant weight changes were reported during follow-up, indicating satisfactory tolerability.

One month after discharge, thyroid function tests (a five-item panel) revealed normalization of all parameters. The patient continued levothyroxine sodium 25 µg daily, with thyroid function monitoring scheduled every three months. It was determined that the current dosage could be maintained provided thyroid function remained within normal limits. Over a six-month follow-up period, the patient's pharyngeal foreign body sensation resolved completely. Repeat thyroid function tests showed TSH at 2.3 mIU/L (normal range: 0.27–4.2 mIU/L) and FT4 at 15.6 pmol/L (normal range: 12.0–22.0 pmol/L), confirming sustained euthyroidism. Serial thyroid ultrasounds at 3 and 6 months post-treatment demonstrated a reduction in the size of the lingual mass to 12×10×14 mm and 10×8×12 mm, respectively, with no significant abnormal vascularity.

Patient Perspective: “As a patient presenting with an unexplained pharyngeal sensation, I was initially anxious about the 'tongue base mass' diagnosis and concerned about potential surgical risks. After starting thyroid hormone replacement, my discomfort gradually subsided. Regular follow-up has provided reassurance, and I am grateful for the accurate diagnosis and individualized management, which avoided unnecessary surgery and enabled me to return to my normal life.”

## 2 METHODOLOGY

For the literature review of this study, the MeSH term “Ectopic Thyroid” was used for a systematic search in the PubMed database, with the search time range from 2000 to 2025. In addition, to supplement the local clinical research data, a supplementary search was conducted for relevant articles in Chinese core journals published in the same time period in the CNKI (China National Knowledge Infrastructure) database. The literature inclusion criteria were as follows: study types including case reports, case series, retrospective cohort studies, systematic reviews, and Meta-analyses, and research topics focusing on the diagnosis, treatment, and prognosis of ectopic thyroid. The literature exclusion criteria were as follows: conference abstracts, unpublished grey literature, duplicate publications, basic research irrelevant to the diagnosis and treatment of ectopic thyroid, and studies with incomplete data from which valid clinical information could not be extracted. Through the above-mentioned search and screening process, a total of 51 Chinese and English literature were finally included in this study. A narrative review was conducted based on the included literature to comprehensively analyze and systematically summarize the research progress in the clinical diagnosis and treatment of ectopic thyroid, which provided evidence-based medical evidence for the diagnostic analysis and discussion of the present case of lingual ectopic thyroid.

## 3 DISCUSSION

### 3.1 Pathophysiology of ET

Normal thyroid tissue in humans is derived primarily from the embryonic endoderm. During the third week of embryonic development, thyroid primordia arise from the normal thyroid gland, which migrates downward from the foramen cecum *via* the thyroglossal duct to the sides and front of the 2nd to 6th tracheal cartilages [2]. Abnormalities are the processes of thyroid primordia migrating from their original location to the final cervical position during embryonic development. Such abnormalities result in the thyroid gland ultimately appearing outside its normal anatomical location, thereby forming ET [3, 4]. Among the sites where ET can occur are the tongue, trachea, lateral cervical region, axilla, carotid artery region, iris, pituitary region, heart, lung, esophagus, duodenum, gallbladder, stomach, pancreas, adrenal gland, ovary, fallopian tube, uterus, and vagina. Most of these ectopic thyroid tissues, however, are located in the neck and at the base of the tongue. As reported in all ET-related literature indexed in PubMed over the past five years, the base of the tongue is the most common site, accounting for approximately 90% of all ET cases [5]. Unclear yet is the molecular mechanism of ET, but studies have suggested that its occurrence may be associated with gene mutations in transcription factors involved in thyroid organogenesis and migration, such as TTF-1, NKX2, Pax-8, FOXE1, and HEX [6, 7]. The above-mentioned transcription factors together constitute the core regulatory network for lineage specification and morphogenesis during the embryonic development of the thyroid gland, and exert an irreplaceable regulatory role in the specification, survival, directed migration, and establishment of epithelial polarity of the thyroid primordium [8, 9]. Among them, the synergistic expression of Nkx2-1 and Pax8 is the core molecular marker of thyroid lineage specification [8, 9]. They not only maintain the survival of thyroid precursor cells by regulating the anti-apoptotic protein Bcl2 [8, 9], but also targetedly regulate the spatiotemporal expression of adhesion molecules such as cadherin 16, thereby modulating the collective migration behavior of thyroid epithelial cells [8, 9]. Foxe1 is a key transcription factor that regulates the detachment of the thyroid primordium from the foramen cecum at the floor of the pharynx and the completion of its physiological caudal migration, and its dysfunction is one of the core molecular mechanisms leading to the arrest of thyroid migration and the development of lingual ectopic thyroid [9]. Hhex participates in the formation and early development of the thyroid bud by regulating the cell cycle progression of thyroid precursor cells, and its aberrant expression can result in thyroid primordium dysplasia and ectopic colonization [8, 9]. Mutations or dysregulation of the expression of the genes encoding these transcription factors will disrupt the ordered process of specification, proliferation, migration and polarization during thyroid organogenesis, ultimately leading to the abnormal colonization of thyroid tissue in non-anatomical sites. This molecularly explains the embryological and genetic basis for the tongue base being the most common site of ET [8, 9].

Clinically, ectopic thyroid is classified based on the presence of thyroid tissue in the normal cervical anatomical location. This classification distinguishes between “aberrant thyroid” (also known as total ectopia) and “accessory thyroid gland”. Aberrant thyroid refers to the absence of the orthotopic gland, with all thyroid tissue located in an ectopic site. In contrast, an accessory thyroid gland describes the presence of a smaller thyroid gland in its normal location, coexisting with the ectopic tissue [10]. The ectopic tissue often serves as the body's only functional thyroid tissue and is frequently associated with hypothyroidism [10].

In the thyroid ultrasound of this patient, no definite thyroid structure was found in the original thyroid area; instead, a slightly hypoechoic nodule, measuring approximately 0.30×0.26 cm with a regular shape and clear boundaries, was detected in the anatomical region of the left thyroid lobe, which can be considered an accessory thyroid.

### 3.2 Clinical Manifestations

Notably, both the present case and published literature reports have revealed a significant gender difference in the incidence of ET. Female patients account for the majority of ET cases, a phenomenon consistent with the epidemiological characteristics of most thyroid diseases. A retrospective study by Santangelo *et al.* of 3092 patients undergoing thyroid surgery showed that among the 28 identified cases of ET, females accounted for as high as 68% [11]. Another case series study of 8 patients with head and neck ET also reported that 5 of the cases (62.5%) were female, further confirming this trend [12]. In a review of relevant literature, the latest case studies also clearly indicated a female majority in the gender distribution of ET, suggesting the existence of potential susceptibility factors [5]. Other studies have also reported a female-to-male incidence ratio of approximately 4:1 [13]. This gender predisposition may be related to the stimulatory effect of the more complex hormonal environment in females on thyroid tissue [14].

Rare is ET in clinical practice, and it lacks typical clinical symptoms; symptoms are usually associated with the size and location of the ectopic tissue as well as related endocrine dysfunction [15, 16]. Predominated by local manifestations are the initial symptoms of patients with ET, among which local masses are the most common, accounting for 73% and mostly occurring in areas such as the oral cavity and neck [17]. The ET can stimulate the organ where it is located, causing symptoms such as pain and discomfort in local tissues; it can also obstruct local organs and induce corresponding symptoms, such as dysphagia, dyspnea, and chest tightness [17, 18]. For instance, reported that ET in the parotid gland region presents as a painless mass under the earlobe that gradually enlarges [7], while ET in the pericardium causes mild chest pain and discomfort [7]. Obvious clinical symptoms are absent in many patients with ET at the base of the tongue, but some women may experience compensatory enlargement during puberty, pregnancy, or menopause, which draws medical attention. The initial symptom of the patient in this case, whose thyroid tissue is ectopic at the base of the tongue, was a foreign body sensation in the pharynx.

### 3.3 Diagnosis

Of great significance for the diagnosis of lingual thyroid are imaging examinations and thyroid function tests. (1) Often chosen as the first-line examination is neck and thyroid B-ultrasound due to its non-invasiveness, convenience, and low cost [7]. Routinely, the mass and the thyroid gland in its normal location should be examined first to determine whether the thyroid gland is in its normal location and whether there are nodules or malignant tumors in the normally located thyroid gland. While B-US can display the size, shape, blood flow signals, and relationship with surrounding structures of the mass, it cannot diagnose the mass as ET; thus, combined with other examinations must be used to confirm whether the mass originates from thyroid tissue [19]. (Fig. **2A**), the thyroid ultrasound, indicated that no definite thyroid structure was detected in the normal thyroid area; only a tiny hypoechoic nodule measuring 0.30×0.26 cm was found in the anatomical region of the left thyroid lobe. This finding supports the diagnosis of an “accessory thyroid gland,” rules out the possibility of “complete ectopic thyroid,” clarifies the type of ectopic thyroid in this case, and lays the foundation for formulating an individualized treatment plan. (2) Useful for identifying the location, size, and relationship with surrounding tissues of ET are CT and Magnetic Resonance Imaging (MRI). Since thyroid tissue is rich in iodine, it usually appears as high density on plain CT scans; on plain MRI scans, it shows relatively high signals on both T1-weighted imaging (T1WI) and T2-weighted imaging (T2WI), slightly high signals on Diffusion-Weighted Imaging (DWI), and high signals on Apparent Diffusion Coefficient (ADC) maps. It is reported that ET may show heterogeneous signals when complicated with canceration, cysts, adenomas, or nodular goiter [3]. In this case, Fig. (**1A**) (fibrolaryngoscopy) showed a semicircular, smooth bulge at the base of the tongue, representing the typical morphological presentation of ectopic thyroid. This can be initially differentiated from the irregular bulge of a tongue malignancy or the diffuse thickening of lingual tonsil hyperplasia. Fig. (**1B**) (neck CT) revealed a mass with clear boundaries, obvious enhancement on contrast-enhanced scan, and surrounding tortuous small blood vessels, consistent with the physiologically rich blood supply of thyroid tissue. Simultaneously, it indicated that the lesion did not cause significant airway narrowing, providing key imaging evidence for the subsequent choice of conservative treatment. (3) Regarded as the optimal method for diagnosing ET is thyroid scintigraphy, as it enables both localization and qualitative diagnosis, distinguishes the relationship between the mass and surrounding blood vessels more clearly, and facilitates diagnosis for clinicians [20]. Also, as high as 95.83% is the accuracy of thyroid scintigraphy in detecting lingual lesions (a common site of ET), which significantly improves the diagnostic efficiency of ET [20]. Fig. (**2B**) (SPECT-CT) showed no iodine uptake in the thyroid bed area, while the lesion at the tongue base demonstrated increased iodine uptake. This constitutes the gold-standard evidence for diagnosing ectopic thyroid, directly confirming the thyroid tissue nature of the lesion, compensating for the limitation of ultrasound in providing a qualitative diagnosis, ultimately clarifying the diagnosis, and preventing misdiagnosis as a non-functional tongue base mass. For patients in whom no thyroid gland is found in the normal cervical thyroid area by B-US, CT, or MRI, but thyroid tissue is detected at the base of the tongue, further thyroid scintigraphy examination should be performed [3]. A small number of ET with low or no function usually do not show imaging signals, so comprehensive judgment based on other imaging findings must be made. (4) For the confirmation of ET, ultrasound-guided Fine-Needle Aspiration (FNA) biopsy can be used. However, the trauma associated with a pathological needle biopsy is prone to causing wound infection; hence, it cannot be used as the first-line method for confirmation [20]. (5) Thyroid function and thyroid autoantibody tests should be routinely conducted for patients with suspected ET. These tests, including Thyroid-Stimulating Hormone (TSH), free triiodothyronine (FT3), FT4, Thyroglobulin antibody (TgAb), and Thyroid Peroxidase Antibody (TPOAb), are essential to assess overall thyroid function and to identify hyperthyroidism, hypothyroidism, or Hashimoto's thyroiditis. However, it cannot distinguish whether the functional abnormality originates from the normally located thyroid gland or ET. Combined with thyroid scintigraphy, the accuracy of diagnosing the source of thyroid dysfunction can be improved [19]. It is reported that approximately 70% of ET patients, especially those with lingual thyroid, are complicated with hypothyroidism; thus, thyroid function tests can also provide certain evidence for the diagnosis of this disease [4, 7]. In this case, a semicircular bulge was found at the base of the tongue during outpatient fibrolaryngoscopy, and the patient was admitted to the hospital with a diagnosis of “tongue base mass”; after completing neck and thyroid B-US, CT, SPECT-CT, and thyroid function tests, the patient was finally diagnosed with lingual thyroid and hypothyroidism, avoiding misdiagnosis.

Based on the multimodal diagnostic strategy detailed above and the clinical characteristics of this case, we constructed a diagnostic and clinical decision-making algorithm for lingual ectopic thyroid, as shown in Fig. (**3**).

### 3.4 Differential Diagnosis

According to statistics by Chinese scholars, the rate of misdiagnosis or unclear diagnosis of lingual thyroid cases reported in China is as high as 63.1%, so attention should be paid to differentiating it from the following diseases [17].

Both lingual thyroglossal duct cysts and lingual thyroid are developmental anomalies related to embryonic development. However, thyroglossal duct cysts are derived from thyroglossal duct remnant tissue, mostly cystic, often located in the midline of the anterior neck and movable with swallowing; lingual thyroid is mostly solid tissue, fixed in the midline area of the tongue base. On thyroid scintigraphy images, thyroglossal duct cysts show no radioactive uptake, while lingual thyroid exhibits specific concentration, and this is the key differentiating point [21].

Lingual squamous cell carcinoma, as a malignant tumor, often presents with symptoms such as pain, blood-tinged sputum, and rapid mass enlargement. Imaging shows the mass invades surrounding tissues, and cancer cells can be detected by a pathological biopsy. In contrast, lingual thyroid mainly presents with compressive symptoms like foreign body sensation in the pharynx and dysphagia; pathological examination reveals normal thyroid follicular tissue with no invasiveness, and some patients are accompanied by thyroid dysfunction. This shows a significant difference from lingual squamous cell carcinoma, which has no thyroid function changes [22].

Lingual tonsil hyperplasia is mostly caused by chronic hyperplasia of lymphoid tissue due to recurrent pharyngeal infections, presenting as diffuse thickening involving both sides of the tongue base. Lingual thyroid, however, is mostly a solitary mass in the midline of the tongue base. On thyroid static scintigraphy, lingual tonsil hyperplasia has no radioactive uptake, while lingual thyroid shows characteristic concentration; additionally, lingual tonsil hyperplasia has no thyroid dysfunction, which is different from lingual thyroid, which is often accompanied by conditions like hypothyroidism [23].

Metastatic thyroid cancer often has a history of primary thyroid cancer, with masses mostly having ill-defined borders and often accompanied by cervical lymph node metastasis. Its thyroid scintigraphy findings differ from those of lingual thyroid, showing no characteristic radioactive concentration. Lingual thyroid, on the other hand, is mostly a solitary mass in the midline of the tongue base with relatively clear borders and no tendency for lymph node metastasis, and thyroid scintigraphy can confirm its thyroid tissue nature [23].

### 3.5 Treatment

The treatment approach for patients with ET is determined based on a comprehensive consideration of factors such as the type, location, size, benign/malignant nature, presence of symptoms, and thyroid function of ET, and an individualized principle should be adopted [7, 19]. For patients with no clinical symptoms and normal thyroid function, clinical intervention is not recommended; however, for patients who have developed clinical symptoms, the treatment plan depends on the location, size, symptoms, nature of the ET gland, and thyroid function. Poor glandular development and hypothyroidism may occur in some cases of ET, which are subsequently followed by compensatory enlargement of the gland. For patients with lingual thyroid who present with clinical symptoms combined with hypothyroidism, oral thyroid hormone administration can be used to inhibit TSH levels. This regulates the body's thyroid hormone levels, normalizes thyroid function, and reduces the volume of the thyroid gland, thereby eliminating the patient's clinical symptoms [4, 15]. The patient in this case had a foreign body sensation in the pharynx and was accompanied by hypothyroidism; after consultation with the Department of Endocrinology and Department of Thyroid Surgery, thyroid hormone replacement therapy was adopted. Levothyroxine sodium is the first-line replacement therapy for patients with ET complicated with hypothyroidism [24-26], and meanwhile, it serves as the preferred conservative treatment for patients with mild benign ET *via* TSH suppression therapy. However, for patients with failed conservative treatment or high-risk symptoms such as severe compression, surgical treatment still holds an irreplaceable core position, and Radioactive Iodine (RAI) therapy is an important alternative for specific benign populations [24].

The absolute indications for surgical treatment include: progressive compressive symptoms caused by the mass, such as severe airway obstruction and dysphagia, recurrent bleeding, cystic changes unresponsive to conservative treatment, and pathologically confirmed or highly suspected malignant transformation. A systematic review by Sauer *et al.* showed that among patients with lingual thyroid, the most common subtype of ET, approximately 86% to 93% of those who received surgical treatment achieved significant improvement or complete resolution of symptoms [27]. With the development of minimally invasive techniques such as Transoral Robotic Surgery (TORS), the complication rate of appropriate surgical approaches has been significantly reduced. Studies have shown that the postoperative bleeding rate of the transoral approach is approximately 4%, and tracheotomy is rarely required, indicating that surgery is a safe and effective treatment for patients who meet the indications [27].

In contrast, RAI is only indicated for patients with benign ET with iodine uptake function. The core applicable population includes patients with surgical contraindications, intolerance to anesthesia, or refusal of surgery, including those with severe underlying diseases such as liver cirrhosis and nephrotic syndrome [28]. Otmani *et al.* reported a case of intratracheal ectopic thyroid causing respiratory distress. In the setting where traditional surgery was not feasible, low-dose ^131^I therapy was administered, after which the intratracheal mass was significantly reduced, and airway obstruction was completely relieved [28]. This case suggests that RAI can be used as an alternative to destroy functional ectopic thyroid tissue and relieve obstructive symptoms in emergency situations where surgery is not feasible; existing clinical studies have also confirmed that RAI is a safe and effective conservative alternative treatment for patients who meet the indications [28]. However, RAI is contraindicated in pregnant and lactating women, patients with suspected or confirmed malignant transformation, patients without iodine uptake function, and those unable to comply with radiation protection. For patients with pre-existing hypothyroidism, RAI is difficult to exert a therapeutic effect due to the extremely poor iodine uptake capacity of ectopic thyroid tissue, and thus is also not recommended [27].

In summary, the selection of treatment strategies for ET requires a strict individualized trade-off based on the presence of functional thyroid tissue in the normal anatomical position of the thyroid gland, thyroid function status, severity of symptoms, risk of malignant transformation, and the overall clinical condition of the patient [27-29].

When patients with lingual thyroid have obvious symptoms such as airway obstruction, sleep snoring, and foreign body sensation in the pharynx, or when the mass is complicated with bleeding, cystic changes, or malignant transformation, surgical treatment may be considered based on the specific situation. Surgical treatments for lingual thyroid can be divided into ET resection and transposition [18].

For ectopic thyroid tumors, careful consideration should be given to symptoms, the type of ET, the benign or malignant nature, as well as the patient's age and thyroid function. The core treatment principle is to preserve thyroid function as much as possible; comprehensive methods, including surgery, thyroid hormone replacement, and iodine radiotherapy, should be used to improve symptoms, prevent and treat hypothyroidism, and lymph node dissection in the corresponding area should be performed if necessary [4, 30, 31].

For patients with lingual thyroid, regardless of the treatment method chosen, close follow-up and observation must be conducted through methods such as ultrasound or thyroid function testing, and corresponding treatments should be administered in a timely manner [6].

### 3.6 Prognosis and Influencing Factors

Closely related to the overall prognosis of ectopic thyroid in the neck and tongue (such as simple ectopic thyroid tissue) are the status of thyroid function, choice of treatment modality, presence of malignant transformation, and whether the ectopic tissue is the only functional thyroid tissue [7]. Most patients have a good prognosis after standardized management.

One of the key factors determining prognosis is the status of thyroid function. When TSH levels increase, it will stimulate the proliferation and enlargement of ET, exacerbate compressive symptoms such as dysphagia and airway obstruction, thereby increasing the difficulty of treatment and worsening the prognosis [23]. If a patient has normal thyroid function and no compressive symptoms, specific treatment is usually not required; only regular follow-up and observation are needed, which basically does not significantly affect the quality of life [21, 23]. For patients without severe obstruction symptoms or with only hypothyroidism, thyroid hormone suppression therapy serves as the first-line conservative treatment, which can effectively alleviate symptoms and improve prognosis; successful conservative treatment can also avoid surgical risks [21, 32, 33].

When conservative treatment is ineffective or severe symptoms such as airway obstruction and bleeding are present, surgical intervention is required, and surgery-related factors directly affect the prognosis. Since ectopic tissue may block the airway, tracheal intubation for anesthesia is relatively difficult; collaboration between the anesthesiology and otolaryngology teams is necessary to reduce intraoperative risks. Completing examinations such as angiography before surgery to clarify the blood supply can reduce intraoperative bleeding and significantly improve surgical prognosis [32, 33].

Of particular note is that most patients with lingual thyroid have no functional thyroid tissue in the normal anatomical location, and the ectopic tissue is the only source of thyroid function [34]. If this condition is not clarified before surgery and the ectopic tissue is blindly resected, permanent hypothyroidism will occur, requiring lifelong levothyroxine replacement therapy. Irregular replacement therapy may also cause problems such as fatigue and metabolic disorders, which seriously affect the prognosis [21, 34]. Therefore, it is crucial to confirm whether the ectopic tissue is the only functional source through radionuclide scanning before surgery; based on this, doctors will prioritize conservative treatment or function-preserving surgical methods to avoid adverse outcomes [34]. However, even if lifelong hypothyroidism occurs due to misdiagnosis and incorrect resection, normal life can still be maintained through standardized hormone replacement therapy [21, 35].

Malignant transformation of ectopic thyroid in the neck and tongue is relatively rare, among which the incidence of lingual thyroid carcinoma is only about 1%, but malignant transformation will significantly worsen the prognosis [22, 36, 37]. Nevertheless, such patients can still achieve good long-term survival after standardized treatment. For cases of malignant transformation, surgical resection is the core of treatment; usually, resection of the ectopic tissue combined with total thyroidectomy is adopted, and some patients require additional radioactive iodine ablation. Long-term and regular postoperative levothyroxine replacement therapy can effectively control the condition and improve the prognosis [22, 38]. A small number of patients may experience recurrence or metastasis in areas such as cervical lymph nodes, but the overall recurrence rate is relatively low [22]. Long-term follow-up results from a single-center retrospective cohort study of 47 patients with ET showed that cases of malignant transformation of lingual ectopic thyroid had no disease recurrence or progression during the follow-up period (mean 59.4 months, maximum 117 months) after surgical resection combined with postoperative TSH suppression therapy. This confirms that although lingual ectopic thyroid has a low risk of malignant transformation, there is indeed a clinical possibility of disease progression to the cancerous stage, and favorable disease control can be achieved after standardized intervention following malignant transformation [30].

Notably, clinical reports have demonstrated that ectopic thyroid tissue can undergo independent malignant transformation in the presence of a normal orthotopic thyroid gland, suggesting that the malignant potential of ectopic thyroid is independent of the presence and functional status of the orthotopic thyroid [39, 40]. For lingual ectopic thyroid that has progressed to the malignant stage, the incidence of regional lymph node metastasis is approximately 20%. Some studies have reported that the overall metastasis rate (including regional and distant) can reach 38%, with a distant metastasis incidence of approximately 14% (the most common sites include the lungs and bones), which are the core factors leading to disease progression and poor prognosis in patients [11, 41].

In terms of disease-related complications, in addition to the local compressive symptoms and permanent hypothyroidism caused by blind surgery mentioned in this study, surgical intervention also carries clear risks of perioperative and long-term complications. In addition, for patients requiring long-term THRT, significantly elevated serum TSH levels (>10 mIU/L) are significantly associated with an increased risk of ischemic heart disease, heart failure, fragility fractures, and all-cause mortality; while excessively low TSH levels (<0.1 mIU/L) are associated with a reduced risk of heart failure, they are still linked to a significantly increased all-cause mortality. Therefore, thyroid function should be monitored regularly during treatment to maintain TSH within the recommended normal range (0.4–4 mIU/L), thereby reducing the risk of systemic complications related to long-term treatment [42].

### 3.7 Study Limitations

Several limitations of this study should be explicitly acknowledged in this section. This study is a single-case report, which only enrolled a 44-year-old female patient with accessory-type lingual ectopic thyroid complicated with mild hypothyroidism. Therefore, the findings can only reflect the diagnosis and treatment experience for patients with this specific subtype, age group, and mild disease severity, and cannot be widely generalized to the entire population with all types of ET. Although the 6-month follow-up period can support the short-term safety and efficacy of levothyroxine sodium therapy, including symptom relief, mass shrinkage, and normalization of thyroid function, this observation period is clearly insufficient for the evaluation of long-term outcomes. In the field of thyroid surgery and endocrinology, 6 months is often regarded as the key time point to distinguish “transient” from “permanent” complications [43]. However, continuous monitoring for several years is required to confirm “no recurrence” or “no malignant transformation” of the disease [30]. In the follow-up, a lifelong standardized follow-up should be conducted for this patient. Meanwhile, large-sample multicenter cohort studies should be conducted to optimize the stratified diagnosis and treatment system, full-dimensional monitoring of prognostic indicators, and surgical risk prevention and control strategies for ET, thereby providing high-level evidence-based medical evidence for the standardized diagnosis and treatment of this disease [42, 44].

## CONCLUSION

This case demonstrates that in patients with unexplained pharyngeal symptoms, a systematic diagnostic approach integrating fibrolaryngoscopy, cross-sectional imaging, thyroid scintigraphy (SPECT-CT), and functional testing is essential for identifying lingual ectopic thyroid and preventing unnecessary surgery; for symptomatic cases with hypothyroidism, initial thyroid hormone replacement therapy effectively normalizes thyroid function, relieves symptoms, and can lead to mass regression, offering a favorable conservative management pathway.

The strength of this report lies in its detailed illustration of a standardized diagnostic workflow and the objective follow-up data supporting the efficacy of levothyroxine therapy, although its generalizability is inherently limited by the single-case design and relatively short follow-up period, highlighting the need for larger, prospective studies to establish long-term outcomes and refine treatment protocols.

## Figures and Tables

**Fig. (1):**
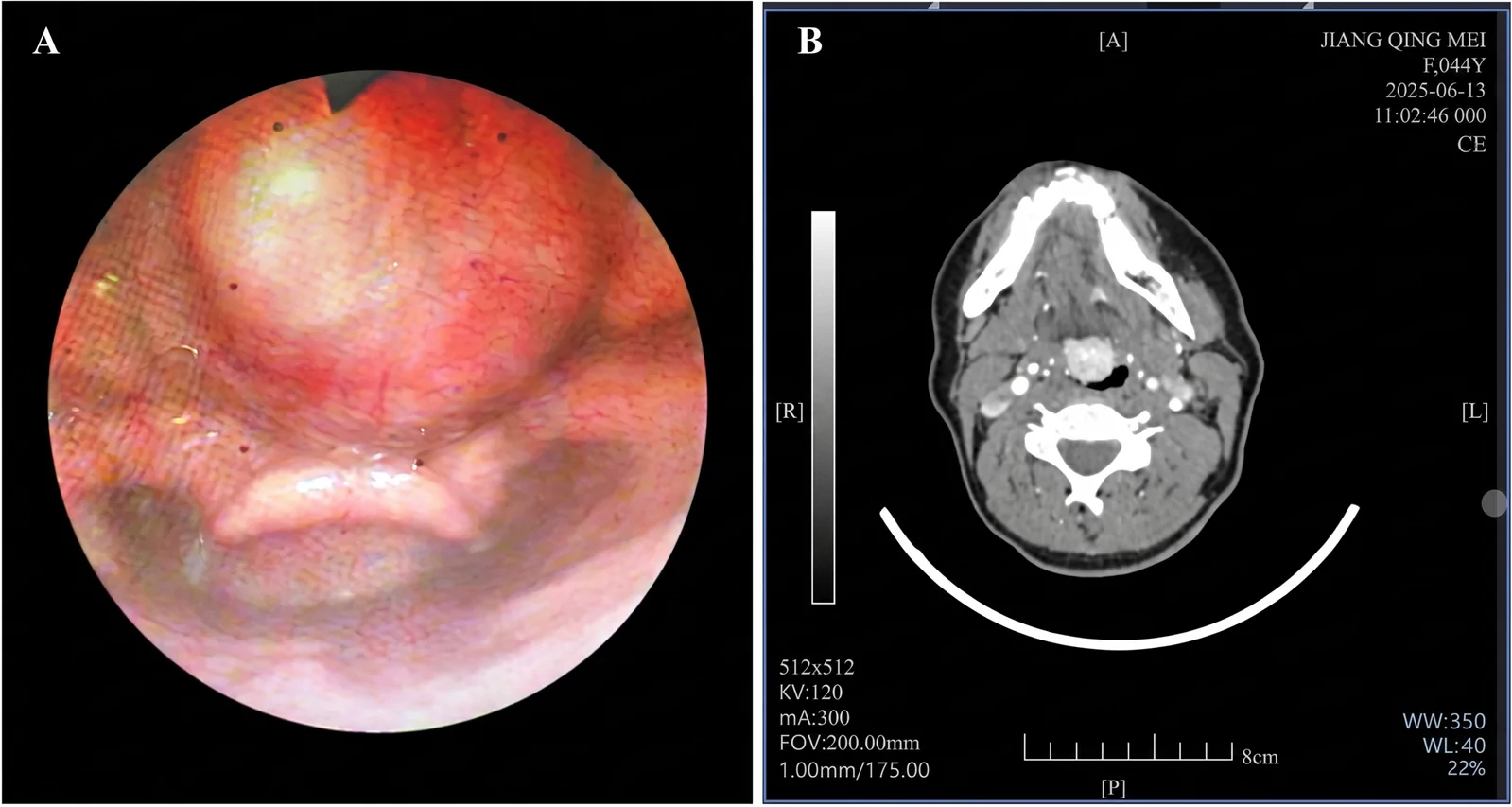
(**A**) Fibrolaryngoscopy showed a semicircular bulge at the base of the tongue with a smooth surface; (**B**) Neck CT revealed an isodense mass shadow in the midline of the tongue base with relatively clear boundaries, showing obvious enhancement on contrast-enhanced scan.

**Fig. (2):**
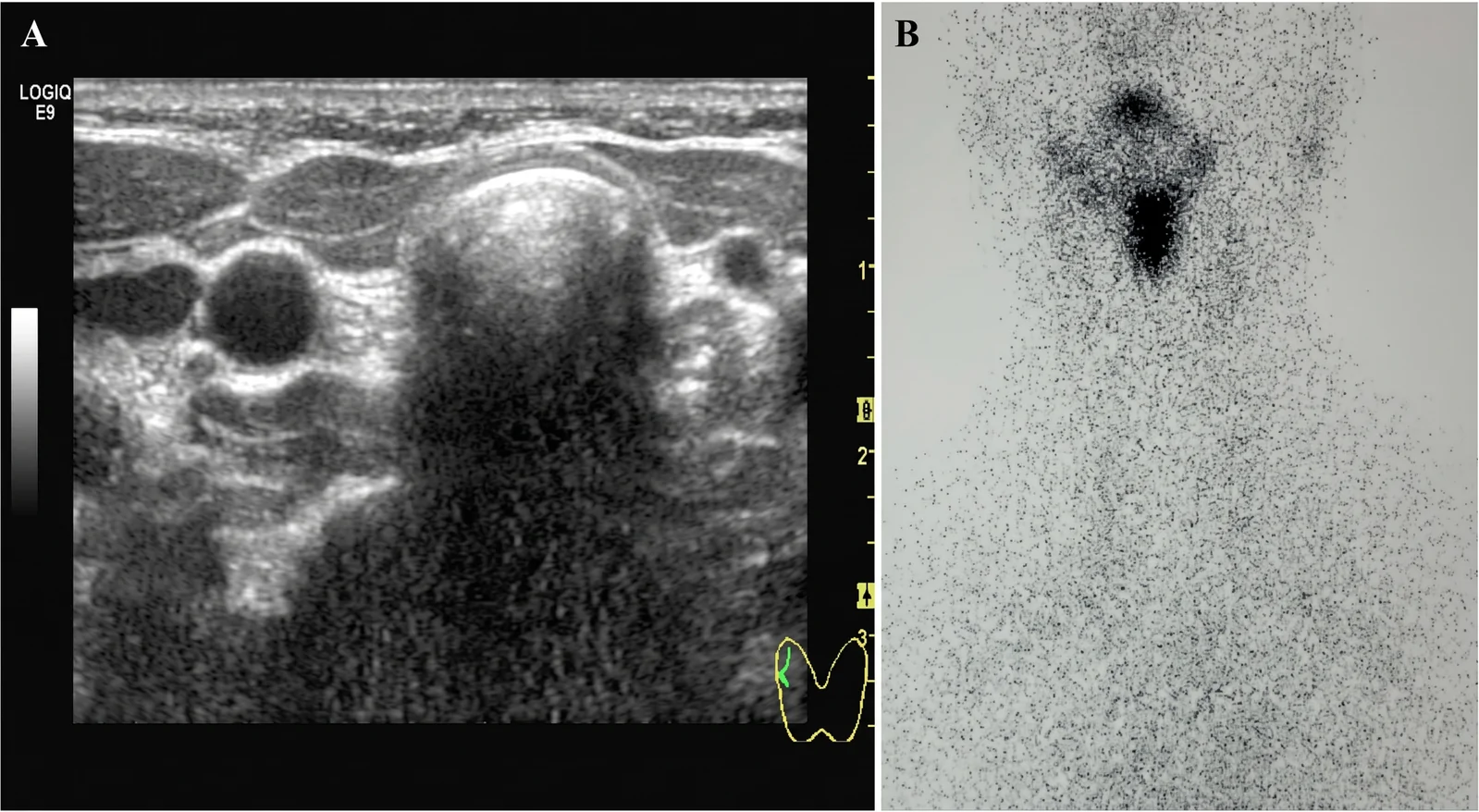
(**A**) Thyroid + cervical lymph node ultrasound showed no definite thyroid structure in the original thyroid area, and a slightly hypoechoic nodule in the anatomical region of the left thyroid lobe; (**B**) Under SPECT-CT images, no thyroid structure was seen in the thyroid bed area, and a patchy, slightly hyperdense shadow was observed in the midline of the tongue base.

**Fig. (3):**
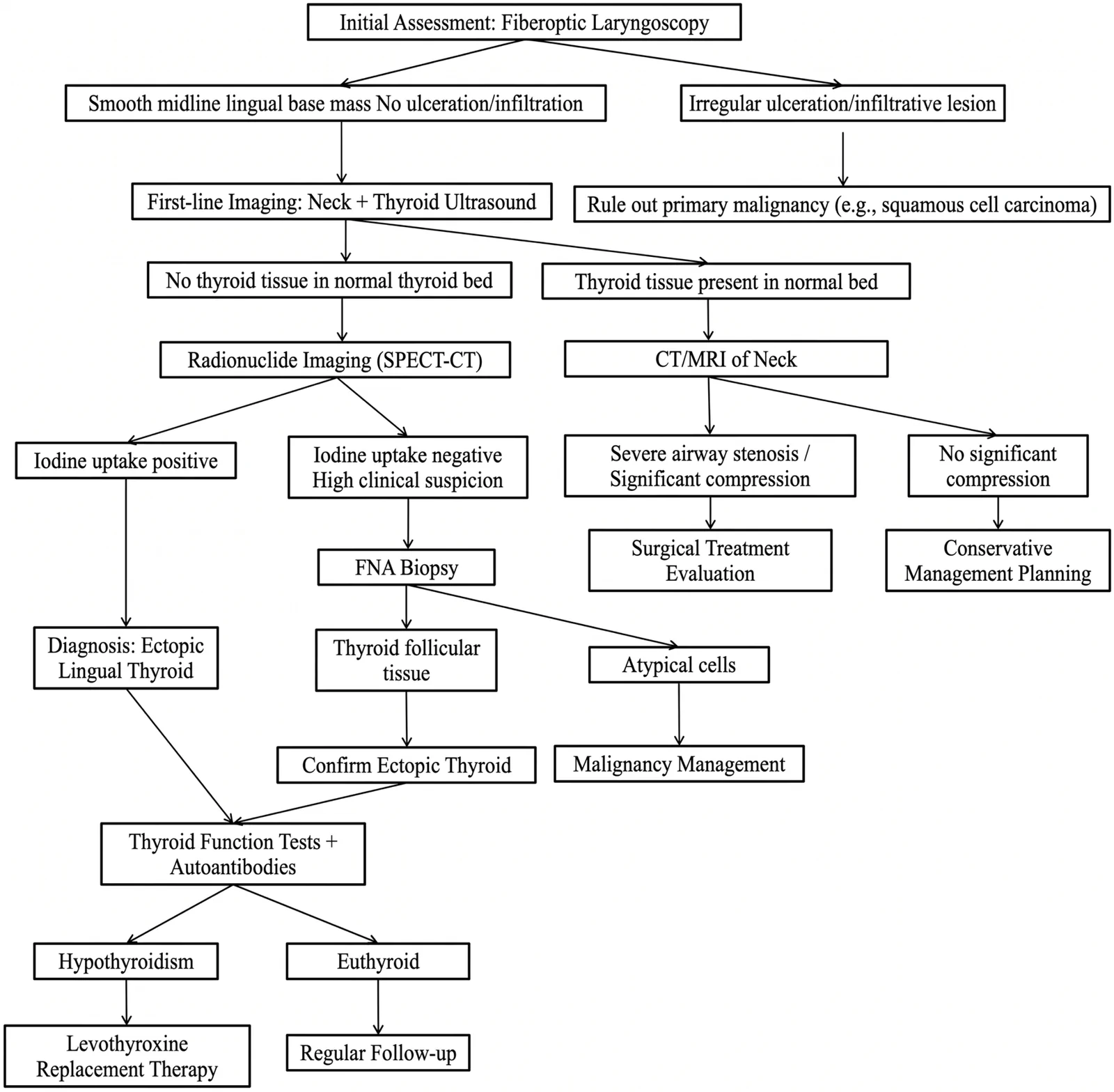
Diagnostic and clinical decision-making flowchart for lingual ectopic thyroid.

## Data Availability

The data that support the findings of this study are not openly available due to ethical restrictions and concerns about patient privacy. De-identified data (excluding personal identifiable information) can be obtained from the corresponding author upon reasonable request. Requests for data access should be directed to guominent@163.com.

## LIST OF ABBREVIATIONS

B-US: B-mode Ultrasound
CARE: Case Report Guidelines
CT: Computed Tomography
ET: Ectopic Thyroid
FT3: Free Triiodothyronine
FT4: Free Thyroxine
Lingual ET: Lingual Ectopic Thyroid
MRI: Magnetic Resonance Imaging
SPECT-CT: Single-Photon Emission Computed Tomography-Computed Tomography
THRT: Thyroid Hormone Replacement Therapy
TSH: Thyroid-Stimulating Hormone
T4: Thyroxine
